# Blood-brain barrier opening-based local delivery of 80 nm-sized liposomes in mice using pulsed focused ultrasound

**DOI:** 10.1186/2050-5736-3-S1-P24

**Published:** 2015-06-30

**Authors:** Zsofia Kovacs, Paola Luciani, Patrick Roth, Jean-Christophe Leroux, Ernst Martin, Beat Werner

**Affiliations:** 1 National Institutes of Health, Bethesda, Maryland, United States; 2 ETH Zurich, Zurich, Switzerland; 3 University Hospital Zurich, Zurich, Switzerland; 4 MR Research Center, Zurich, Switzerland; 5 University Children’s Hospital Zurich, Zurich, Switzerland

## Background/introduction

Continuous focused ultrasound (FUS) has been mostly used to generate high temperatures necessary for tissue ablation. Pulsed FUS in combination with intravenously injected microbubbles has been shown to open the blood-brain barrier (BBB) to increase plasma-to-tissue permeability, thus presenting a new opportunity for local drug delivery to the targeted part of the brain. We have previously demonstrated a local delivery of doxorubicin to mouse brain tumor tissues by transient BBB opening.[1] However, the next generation of therapeutics is associated with a broader range of clinically meaningful applications. Liposomes are particularly interesting since drug and contrast agent can be combined in a single safe and biodegradable system with a sustained release of content. Therefore, we investigated the brain delivery of fluorescently labeled liposomes by FUS-mediated BBB opening in healthy mice.

## Methods

The 80 nm-sized vesicles were either labeled with cholesteryl 4,4-difluoro-5,7-dimethyl-4-bora-3a,4a-diaza-s-indacene-3-dodecanoate (chol-BODIPY^®^) incorporated into the lipid bilayer or both with BODIPY^®^ and an additional dye, whose crosstalking with BODIPY^®^ was ruled out (IRDye^®^ 680LT and ROX™) and was conjugated to the liposome surface. The systemically injected liposomes (100 μL) were washed out of the bloodstream 2 h after sonication. FUS was created by a single-element, spherical FUS transducer (center frequency: 612.5 kHz; focal depth: 50 mm; active diameter: 64 mm; model: H-107_MR, Sonic Concepts, Bothell, WA). All FUS sonications started 30 s after the injection of 60 μL lipid-coated gas microbubbles via the tail vein in B6 (Cg)-Tyrc-2J/J mice (n=8).

## Results and conclusions

**Figure 1:**
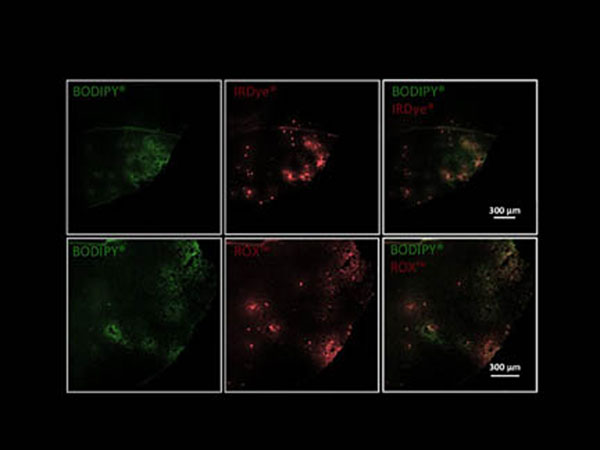
Stereomicroscopy images of horizontal mouse brain section showing identical extravasation of BODIPY^®^ and IRDye^®^ 680LT as well as BODIPY^®^ and ROX™ at the sonication site.

Selective and efficient delivery of long-circulating fluorescent liposomes could be achieved by optimal acoustic parameters: 612.5 kHz, 0.4 MPa for a 4 min duration in bursts of 10 ms length at 1 Hz repetition time. In the case of dually-labeled liposomes the IRDye^®^ 680LT and ROX™ showed co-localized extravasation pattern with BODIPY^®^ on the mouse brain sections (Figure 1), which indicates intact liposomes at the sonication site. This demonstration of liposome delivery across the BBB via FUS has opened new scenarios to deliver relevant therapeutics up to 80 nm in size to the brain. Furthermore, it may represent the basis for other therapeutic directions involving pulsed or continuous FUS sonications.
